# Supplementary material for: In Vitro Effects of Cannabidiol on Activated Immune–Inflammatory Pathways in Major Depressive Patients and Healthy Controls
Source: Pharmaceuticals (Basel). 2022 Mar 26;15(4):405. doi: 10.3390/ph15040405 (PMC9032852; doi:10.3390/ph15040405)
Supplement: Supplementary file 1 [file pharmaceuticals-15-00405-s001.zip › pharmaceuticals-1624955-supplementary.pdf]

**ELECTRONIC SUPPLEMENTARY FILE**

# Effects of cannabidiol on activated immune-inflammatory pathways in major depressive patients and healthy controls.

(1) Muanpetch Rachayon; (1,2) Ketsupar Jirakran; (3) Pimpayao Sodsai; (4) Siriwan Klinchanhom; (1) *Atapol Sughondhabirom*; (5) Kitiporn Plaimas; (6) Apichat Suratanee; (1,7,8) Michael Maes

(1) Department of Psychiatry, Faculty of Medicine, Chulalongkorn University and King Chulalongkorn Memorial Hospital, the Thai Red Cross Society, Bangkok, Thailand

(2) Maximizing Thai Children's Developmental Potential Research Unit, Department of Pediatrics, Faculty of Medicine, Chulalongkorn University, Bangkok, Thailand

(3) Center of Excellence in Immunology and Immune-Mediated Diseases, Department of Microbiology, Faculty of Medicine, Chulalongkorn University, Bangkok, Thailand

(4) Division of Immunology, Department of Microbiology, Faculty of Medicine, Chulalongkorn University, Bangkok, Thailand

(5) Advanced Virtual and Intelligent Computing (AVIC) Center, Department of Mathematics and Computer Science, Faculty of Science, Chulalongkorn University, Bangkok 10330, Thailand

(6) Department of Mathematics, Faculty of Applied Science, King Mongkut's University of Technology North Bangkok, Bangkok 10800, Thailand

(7) IMPACT Strategic Research Center, Barwon Health, Geelong, Australia;

(8) Department of Psychiatry, Medical University of Plovdiv, Plovdiv, Bulgaria

**Table S1.** Cytokine, chemokines and growth factors examined in the current study

| Protein abbreviations | Protein name | Gene Symbol | Number of samples that lower than sensitivity of the assay |
|---|---|---|---|
| IL-1β | Interleukin-1β | IL1B | 0 |
| IL-1RA | Interleukin-1 receptor antagonist | IL1RN | 0 |
| IL-2 | Interleukin-2 | IL2 | 0 |
| IL-4 | Interleukin-4 | IL4 | 0 |
| IL-5 | Interleukin-5 | IL5 | 0 |
| IL-6 | Interleukin-6 | IL6 | 0 |
| IL-7 | Interleukin-7 | IL7 | 125 (out of 200) |
| CXCL8 / IL-8 | C-X-C motif chemokine ligand 8 | CXCL8 | 0 |
| IL-9 | Interleukin-9 | IL9 | 0 |
| IL-10 | Interleukin-10 | IL10 | 0 |
| IL-12 | Interleukin-12 | IL12 | 1 |
| IL-13 | Interleukin-13 | IL13 | 60 (out of 200) |
| IL-15 | Interleukin-15 | IL15 | 0 |
| IL-17 | Interleukin-17 | IL17 | 0 |
| CCL11 | Eotaxin | CCL11 | 0 |
| FGF2 | Fibroblast growth factor 2, Basic fibroblast growth factor | FGF2 | 0 |
| G-CSF | Granulocyte Colony Stimulating Factor, Colony Stimulating Factor 3 (Granulocyte) | CSF3 | 0 |
| GM-CSF | Granulocyte-macrophage colony-stimulating factor, Colony-stimulating factor 2 | CSF2 | 0 |
| IFN-γ | Interferon-γ | IFNG | 0 |
| CXCL10 /IP-10 | C-X-C motif chemokine ligand 8, Interferon gamma-induced protein 10 | CXCL10 | 0 |
| CCL2 / MCP1 | C-C Motif Chemokine Ligand 2 | CCL2 | 0 |
| CCL3 / MIP-1α | Macrophage inflammatory protein-1 alpha, C-C Motif Chemokine Ligand 3 | CCL3 | 0 |

| | | | |
|---|---|---|---|
| PDGF | Platelet Derived Growth Factor Subunit B | PDGFB | 0 |
| CCL4 / MIP-1β | C-C Motif Chemokine Ligand 4, Macrophage Inflammatory Protein 1-Beta, Lymphocyte Activation Gene 1 Protein | CCL4 | 0 |
| CCL5 /RANTES | C-C Motif Chemokine Ligand 5, Regulated Upon Activation, Normally T-Expressed, And Presumably Secreted | CCL5 | 0 |
| TNF-α | Tumor Necrosis Factor-Alpha | TNF | 0 |
| VEGF | Vascular Endothelial Growth Factor | VEGFA | 0 |

**Table S2**. Description of the immune profiles used in this study

| Immune Profile | Members |
|---|---|
| **M1 macrophage** | IL-1β, sIL-1RA, IL-6, TNF-α, CXCL8, CCL3 |
| **T helper-1** | IL-2, IFN-γ, IL-12 |
| **T helper-2** | IL-4, IL-5, IL-9, IL-13 |
| **T helper-17** | IL-6, IL-17 |
| **IRS** | IL-1β, IL-6, TNF-α, CXCL8, CCL3, IL-2, IFN-γ, IL-12, IL-17, IL-15, G-CSF, GM-CSF, CXCL10, CCL5, CCL2 |
| **CIRS** | IL-4, IL-10, sIL-1RA |
| **Neurotoxicity** | IL-1β, IL-6, TNF-α, CXCL8, CCL3, IL-2, IFN-γ, IL-12, IL-17, CXCL10, CCL11, CCL5, CCL2 |
| **T cell growth** | IL4, IL9, IL12, GM-CSF, IL-15 |
| **Growth factors** | FGF, PDGF, VEGF |

IRS: immune-inflammatory response system; CIRS: compensatory immunoregulatory system

## GM-CSF¶

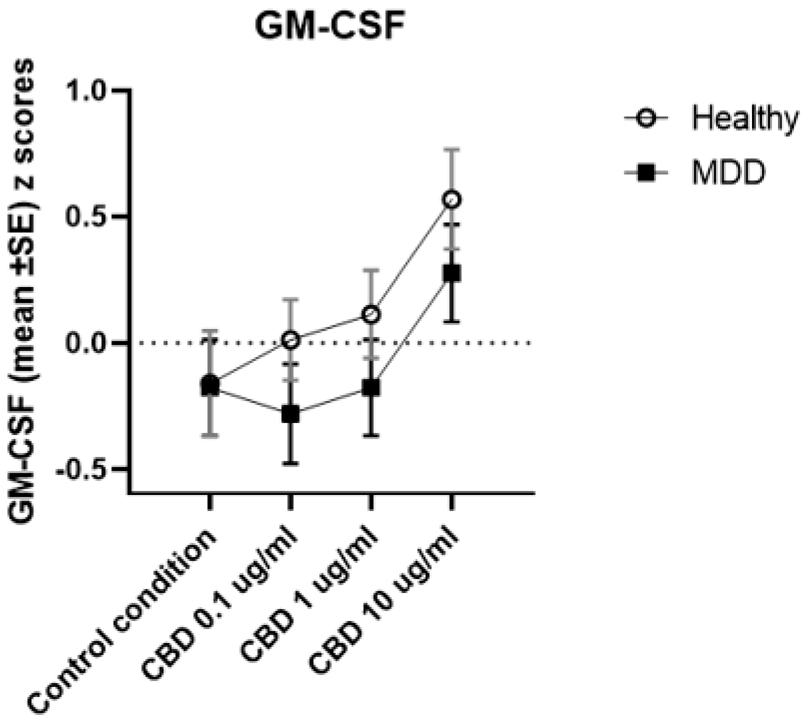


**Figure S1**. Effects of the Interaction between diagnosis and cannabidiol (CBD) administration on the production of granulocyte-macrophage colony-stimulating factor (GM-CSF) in culture supernatant

CCL5·(RANTES)¶

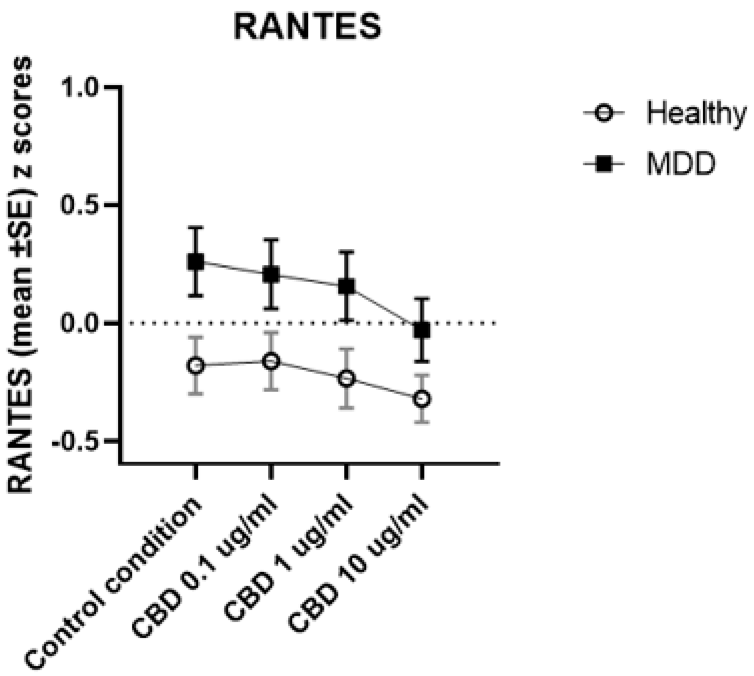


**Figure S2**. Effects of the Interaction between diagnosis and cannabidiol (CBD) administration on the production of CCL5 in culture supernatant

## CXCL8·(IL-8)¶

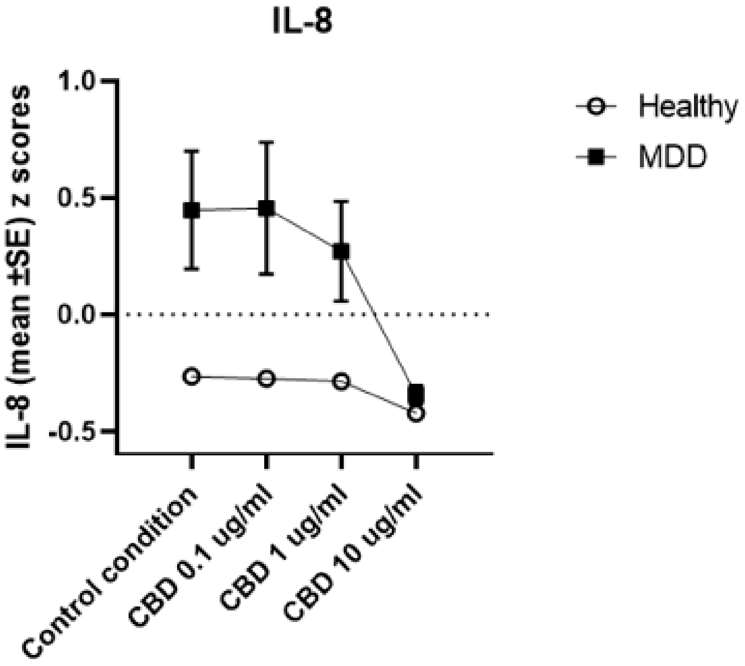


**Figure S3**. Effects of the Interaction between diagnosis and cannabidiol (CBD) administration on CXCL8 production in culture supernatant
